# Molecular identification and evolutionary relationships between the subspecies of *Musa* by DNA barcodes

**DOI:** 10.1186/s12864-020-07036-5

**Published:** 2020-09-24

**Authors:** S. Dhivya, S. Ashutosh, I. Gowtham, V. Baskar, A. Baala Harini, S. Mukunthakumar, R. Sathishkumar

**Affiliations:** 1 Plant Genetic Engineering Laboratory, Department of Biotechnology, Bharathiar University, Coimbatore, 641046 India; 2 Technologico de Monterrey, Centre of Bioengineering, Epigmenio Gonzalez #500, Fracc. San Pablo, Campus Queretaro, Santiago de Querétaro, Queretaro Mexico; 3 Plant Biofarming Laboratory, DRDO-BU Centre for Life Sciences, Bharathiar University, Coimbatore, 641046 India; 4 Biotechnology and Bioinformatics Division, Jawaharlal Nehru Tropical Botanic Garden & Research Institute, Palode, Thiruvananthapuram, Kerala 695 562 India

**Keywords:** *Musa* sp., Cultivar, Wild species, DNA barcode, Nucleotide diversity, Genetic relationship

## Abstract

**Background:**

The banana (*Musa* sp., AAA) genome is constantly increasing due to high-frequency of somaclonal variations. Due to its large diversity, a conventional numerical and morphological based taxonomic identification of banana cultivars is laborious, difficult and often leads to subject of disagreements.

**Results:**

Hence, in the present study, we used universal DNA barcode ITS2 region to identify and to find the genetic relationship between the cultivars and varieties of banana. Herein, a total of 16 banana cultivars were PCR amplified using ITS2 primer pair. In addition, 321 sequences which were retrieved from GenBank, USA, were used in this study. The sequences were then aligned using Clustal W and genetic distances were computed using MEGA V5.1. The study showed significant divergence between the intra- and inter-specific genetic distances in ITS2 region. BLAST1 and Distance methods proved that ITS2 DNA barcode region successfully identified and distinguished the cultivar and varieties of banana.

**Conclusion:**

Thus, from the results of the present study, it is clear that ITS2 is not only an efficient DNA barcode to identify the banana species but also a potential candidate for enumerating the phylogenetic relationships between the subspecies and cultivars. This is the first comprehensive study to categorically distinguish the economically important banana subspecies and varieties using DNA barcodes and to understand its evolutionary relationship.

## Background

Banana and plantain belong to the family *Musaceae* and are cultivated throughout tropical and subtropical regions of the world [1]. This is an important crop next to rice, wheat and corn [2]. The edible *Musa* species and their hybrids and polyploids originated from the two main wild species of banana, viz., *Musa acuminata* Colla and *M. balbisiana* Colla, with A and B genomes, respectively [3]. The major cultivars belong to the subgroups of Cavendish (AAA), Lujugira (AAA), Figue Pomme (AAB), Plantain (AAB), Saba Bluggoe (ABB) and Sucier (AA) [4]. Banana is a staple edible fruit crop with a good source of potassium and magnesium, which provides health benefits, such as, maintaining normal blood pressure and protecting against heart ailments [5]. The genome is continuously expanding due to the occurrence of high frequency somaclonal variation, increasing diversity, leading to quite often subject of disagreements [6].

There have been extensive discussions related to the identities of the progenitors of domesticated banana. *M. acuminata* and *M. balbisiana* have been proposed as wild parents of modern banana. There are four wild species, viz., *M. acuminata* (donor of A genome), *M. balbisiana* (donor of B genome), *M. schizocarpa* (donor of S genome) and *M. textilis* (donor of T genome), which have contributed to the gene pool of bananas. These wild species are extensively distributed in the subtropical and tropical regions of Asia. There are nine subspecies, such as, *banksii, burmannica, burmannicoides, errans, malaccensis, microcarpa, siamea, truncata* and *zebrina* are which are identified as offspring of *M. acuminata* X *M. balbisiana* and these hybrids are found to exhibit several commonly occurring morphological characters. Though the species *M. schizocarpa* and *M. textilis* were endemic to Papua New Guinea, they do not show morphological diversification [7]. The species *Musa nagensium* remained unnoticed by botanists for quite long time and no collections were made more than a century from the habitat, viz., North-East India [8]. In addition, literature shows that the species *M. cheesmanii N.W. simmonds* was misidentified as *M. nagensium* and was provided with the photograph of the former. It was subsequently rediscovered and detailed description was provided by both [9, 10]. Similarly, there was a misidentification of a species with AAA genome, which showed similarity to ABB genome [8]. The occurrence of genetic diversity has been satisfactorily documented in the pool of *M. acuminata* species by employing primers of highly repetitive sequences and tandem repeats by [11, 12]*.* The total number of cultivars in bananas and plantains was estimated to be around 300–1000 and their nomenclature and descriptions are found to be highly ambiguous even within a country [13]. Cultivated bananas are found to differ markedly from their wild relatives due to multiplication through vegetative propagation, exhibiting a high level of morphological diversification [7]. Accordingly, later formulated classification schemes were found to be unambiguous and coherent and were accepted widely. Though the cultivated banana has socio-economic importance, genetic studies are found to be limited in this flora due to the occurrence of extensive polyploidy and parthenocarpy together with complexity associated with sample collection protocols. It is realized that correct identification of *Musa* cultivars is crucial for utilization of this crop species and also important for conservation of the genetic resources. Traditional methods to identify *Musa* cultivars relies more on morphological characters [14] which are often affected by environmental and developmental factors. Phenotypic classification and their usage in inferring genetic relationships among the different genotypes are still under debate [7]. Even employment of molecular markers, such as, Random Amplified Polymorphic DNA (RAPD) [15] did not provide sufficient discriminating capacity for classifying the nine genotypes of *Musa* [16]. Genomic in situ hybridization (GISH) is also not found to be suitable for high-throughput screening of large breeding populations [17]. It is pertinent to mention that DNA markers were employed for identifying dwarf Cavendish banana derived from micropropagation [18]. DNA fingerprinting methodologies are found to be useful in detecting the relationship between parental genotypes with progeny populations [19]. Plastid subtype identity (PS-ID) through sequence analysis was carried out to show the possible maternal relationship among *Musa* sp. [20]. Polymerase chain reaction-restriction fragment length polymorphism (PCR-RFLP) markers of ribosomal internal transcribed spacers (ITS) were used to determine the *Musa* genome and hybrids at the nursery stage [21]. With the current status of research in this area of foolproof identification of *Musa* species and cultivars, a simple and accurate method is highly required for determining the genetic variation between the different cultivars of *Musa* species.

DNA barcoding is a recent technique that uses short and standardized DNA fragments to discriminate the specimens at the species level [22–24]. Many disputed species have been correctly identified by employing DNA barcoding [25]. Herbal products have also been authenticated through DNA barcodes [26, 27]. A few studies have reported the applicability of DNA barcodes even for identifying herbarium samples [28], intra-specific ecotypes [29] and ornamental species for horticultural industries [30]. Hence, DNA barcoding has become an efficient tool for identification with discriminating power at the species level [23]. The chloroplast DNA sequences, such as, *matK, rbcL, psbA-trnH,* and *atpF-atpH* and internal transcribe spacer (ITS) region of nuclear ribosomal DNA have been proposed as potential plant barcodes [31]*.* The internal transcribed spacer 2 (ITS2) is located between the ribosomal 5.8S and 28S, which is actively involved in the regulation of the transcription of active ribosomal subunits and it is essential for pre-rRNA processing [32]. By employing the conserved regions, it is easy to design a universal primer, PCR amplification and DNA sequencing of amplicons which will reveal the variability that can be used to distinguish the closely related species. Due to this universality, in the current scenario, ITS2 has been considered to be a standard barcode for authenticating different medicinal plants [33–36]. Recently DNA barcoding has been appropriately employed in clear identification of the different varieties of plants, imported teas [37] and small millet land races [38]. In the present study, DNA barcoding analysis was performed for the banana cultivars and wild *Musa* accessions using the internal transcribed spacer region ITS*2* for a better understanding of the origin and domestication of cultivated banana and to clear the confusions that exist in the nomenclature and varietal synonyms.

## Results

### PCR success rate and DNA sequencing

The amplification and sequence success rate of the ITS2 sequences from sampled specimens of *Musa* sp. was found to be 100%. The lengths of the ITS2 sequences used for the analyses were in the range of 325–375 bp, with an average of 345 bp. The mean GC content was 60.3%, with a range of 58.3–69.0%.

### Genetic diversity

Genetic divergences were estimated using six metrics, such as, average inter-specific distance, the minimum inter-specific distance, theta prime, average intra-specific distance, coalescent depth and theta. The region ITS2 exhibits significant divergences at the inter-species level (Table 1) at the level of cultivars and varieties. At the intra-specific level, relatively lower divergences were observed for all the corresponding metrics.

**Table 1:** Genetic distance determination using ITS2 region for 277 samples of 46 *Musa* cultivars

Measurement	Kimura 2- parameters (K2P) value
All inter-specific distance	0.194 ± 0.076
Theta prime	0.187 ± 0.064
The minimum inter-specific distances	0.122 ± 0.046
All intra-specific distances	0.035 ± 0.006
Theta	0.081 ± 0.004
Coalescent depth	0.070 ± 0.018

### Assessment of barcoding gap

**Fig. 1:**
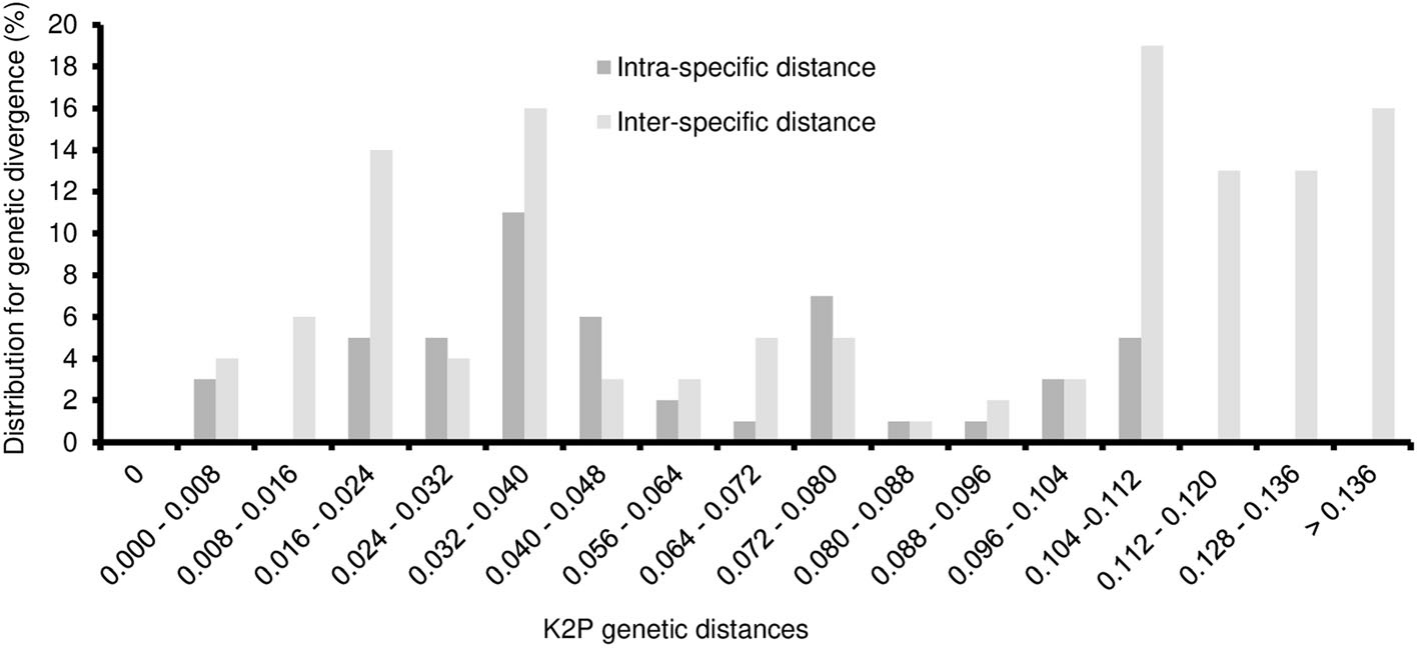
The Relative distribution of inter-specific divergence between congeneric *Musa* species and intra-specific variation (*P* < 0.001) by MEGAX

inter-specific versus intra-specific divergence was analyzed by examining the distribution of genetic distance at a scale of 0.008 distance units. Observations showed that there was only a slight overlap in inter and intra-specific variation (Fig. 1). The inter-specific distance was found to be in the range of 0.002–0.184 equaled 0.002 for only 0.26% and the proportion of inter-specific genetic distance < 0.135 was about 8.33%. The intra-specific distance ranged from 0.000 to 0.135, and most *Musa* species with more than two samples in our study had a unique sequence (58.93%) in the ITS2 region. The results indicated the existence of significantly evident barcoding gap between inter and intra-specific divergence thus indicating that ITS2 could provide a useful region to identify and authenticate different *Musa* species.

### Efficacy of ITS2 for authentication

ITS2 showed 97.7 and 95.8% identification success rates at the species level for the 321 analysed samples of *Musa* using BLAST1 and nearest genetic distance, respectively. Nearly 15 cultivar and wild species were identified that are shown in Table 2. Thus, the ITS2 region exhibited high identification efficiency for *Musa* sp.

**Table 2:** Identification efficiency for ITS2 using BLAST1 and DISTANCE methods

Sample Name	Sample Identified	Methods of Identification	Correct Identification (%)	Ambiguous Identification (%)
*Robusta*	*M. aurantiaca*	*BLAST1*	97	3
		*DISTANCE*	97.06	2.94
*Nadan*	*M. banksii*	*BLAST1*	96	3
		*DISTANCE*	96.04	3.06
*Wild Musa accuminatavar.burmanicoides AA*	*Musa ABB*	*BLAST1*	99	1
		*DISTANCE*	99.7	0.3
*Wild Musa accuminata AA*	*M. acuminata* subsp. *malaccensis*	*BLAST1*	99	1
		*DISTANCE*	99.37	0.63
*Njalipoovan AB*	*Musa acuminata* subsp. *malaccensis*	*BLAST1*	98	2
		*DISTANCE*	98.45	1.55
*Wild Musa balbisiana BB*	*Musa laterita*	*BLAST1*	98	2
		*DISTANCE*	98.41	1.59
*Wild Musa balbisiana BB*	*Musa balbisiana*	*BLAST1*	98	2
		*DISTANCE*	97.68	2.32
*Wild Musa accuminata*	*Musa accuminata*	*BLAST1*	96	4
		*DISTANCE*	95.81	4.19
*Cultivar Pisang lilin*	*Musa acuminata var. zebrina*	*BLAST1*	99	1
		*DISTANCE*	98.9	1.1
*Cultivar Chemmatti - AA*	*Musa campestris*	*BLAST1*	97	3
		*DISTANCE*	96.7	3.3
*Wild Musa accuminata*	*Musa laterita*	*BLAST1*	95	5
		*DISTANCE*	95.15	4.85
*Cultivar Kunnan BA*	*Musa acuminata* subsp. *malaccensis*	*BLAST1*	99	1
		*DISTANCE*	99	1
*Grandnain*	*Musa acuminata*	*BLAST1*	94	6
		*DISTANCE*	94.11	5.89
*Cultivar Matti AA*	*Musa acuminata var. zebrina*	*BLAST1*	99	1
		*DISTANCE*	98.21	1.79
*Red Banana*	*Musa acuminata* subsp. *malaccensis*	*BLAST1*	99	1
		*DISTANCE*	99.1	0.9

### Sequence analysis and species discrimination

**Fig. 2:**
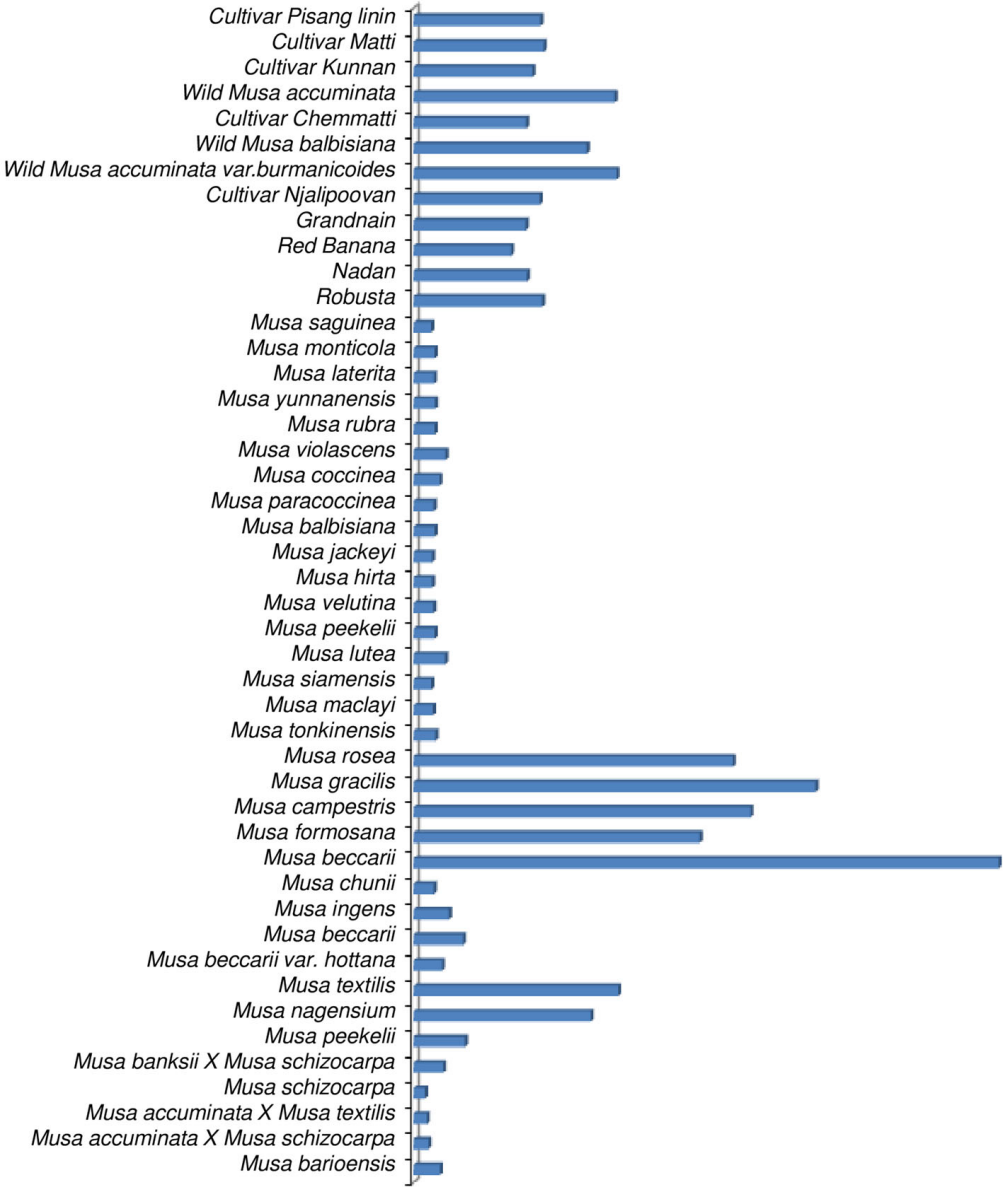
The heterogeneity of individual taxa by ITS2 based on 46 *Musa* subspecies by MEGAX. The left side shows the complete list of *Musa* species used in this study. The right side depicts heterogeneity between and within species, where the values are calculated by using a similarity matrix for biomarker ITS2 with different OTUs (Operational Taxonomic Units)

ITS2 sequences were collected and evaluated using MEGA (Fig. 2). As a result, over 95.6% of species had larger inter-than intra-specific diversity; therefore, there were relatively clear species boundaries for ITS2 sequences. Only two species which were found to fall under the exception category, viz., *M. schizocarpa* and *M. acuminata* x *M. textilis* had very less variability of about 0.035%. ITS2 region showed higher polymorphic sites representing higher genetic diversity in between subspecies and cultivars of *Musa.* Unique haplotypes of *Musa* species and subspecies were identified by using restriction enzymes, such as, *MseI, Pst*I and *Ava*II*,* respectively and are shown in Table 3.

**Table 3:** Haplotypes discrimination of ITS2 *sequence* using Restriction Enzyme Mapping

Species Name	*MseI*	*Pst*I	*Ava*II
*Robusta*	363	–	293, 324
*Nadan*	63	–	333, 353
*Wild Musa accuminata var.burmanicoides AA*	50	32	88, 119, 139
*Wild Musa accuminata AA*	28	–	84, 97
*Njalipoovan AB*	–	–	35, 66
*Wild Musa balbisiana BB*	5444	–	354, 374
*Wild Musa balbisiana BB*	5		354, 374
*Wild Musa accuminata*	11	–	111
*Cultivar Pisang*	370	–	280, 300
*Cultivar Chemmatti - AA*	54, 411	–	321, 341, 372
*Wild Musa accuminata*	28	–	84, 97
*Cultivar Kunnan BA*	148	–	186, 217, 237
*Grandnain*	107, 116	–	–
*Cultivar Matti AA*	77	–	115, 146
*Red Banana*	353	–	283, 314

### Nucleotide polymorphism and neutrality tests

DNA polymorphism analysis showed rich genomic variations in *Musa* accessions, with the total number of polymorphic sites being 112 in cultivated bananas in A genome and 33 in *B* genome. Nucleotide diversity (π and θ) for all the cultivated and wild *Musa* accessions were estimated for silent, non-synonymous and total sites independently. Summaries of nucleotide diversity data for two ITS2 regions are given in Table 4. Reduced levels of polymorphism emerged as a general property of cultivated bananas as compared to their wild progenitors. It shows that subspecies has slightly higher level of nucleotide diversity than wild and cultivated species. Thus, these findings suggest that the cultivars would not have undergone any severe genetic bottleneck during the initial domestication process. The triploid genome AAA and AAB groups also hold high levels of nucleotide diversity, representing the historical population sizes are large. The ABB genome of cultivated banana shows higher nucleotide diversity than that of *M. balbisiana* (Table 4). We found that nucleotide diversity at non-synonymous sites ITS2 region was reduced in the A genome of wild species represented as shown in Table 4. No polymorphic sites were observed within the cultivar and subspecies. However, it was found that the genetic diversity of the AAA genome was 4-6 and folds higher than A genome cultivars. Additionally, the patterns of nucleotide variations in ITS2 region was examined for deviation from neutral equilibrium evolution using Tajima’s neutrality (D) test. Thus, the observations of the present study showed no significant departure from the neutral model.

**Table 4:** Summary of nucleotide diversity and neutrality test statistics for ITS2

Taxonomic groups	Genome	Ha	S	Ps	Θ	ℼ	D
A genome	AAA	39	283	0.632	0.531	0.531	−0.18425
	AAB	8	75	0.115	0.076	0.074	0
	AA Wild Species	4	16	0.089	0.049	0.049	0.123
	A Subspecies	13	132	0.589	0.189	0.73	−0.833
	A Cultivar	48	132	0.663	0.149	0.113	−0.845
	Over All	112	638	0.417	0.198	0.299	− 0.347
B genome	BB	6	23	0.125	0.054	0.052	−0.235
	ABB	27	233	0.373	0.125	0.120	−0.5
	Over All	33	263	0.498	0.179	0.172	−0.735

### Phylogenetic analysis

**Fig. 3:**
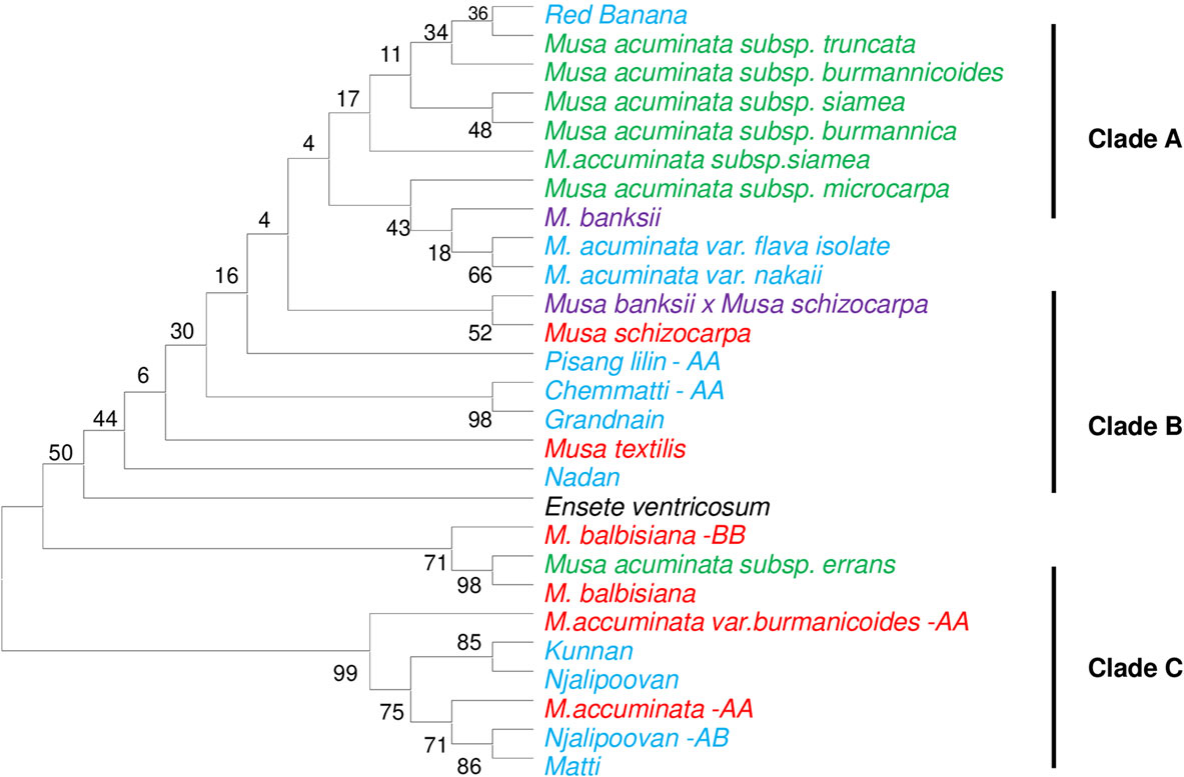
Maximum Likelihood (ML) tree for *Musa* accessions using the ITS2 region. Numbers are bootstrap percentage above 50%. Capital letters following each accession name indicate the previously- recognized genome composition of the cultivar. The appearance of an accession more than once represents a distinct sequence cloned from the same cultivar. Red indicates wild species, green indicates subspecies and blue indicates cultivar

The molecular classification of *Musa* species is based on DNA based profiling [39, 40]. To analyze the phylogenetic relationship of *Musa* cultivars with wild species, nearly 103 species representing 60 cultivars, 5 wild species and 9 subspecies were studied using the Maximum Likelihood (ML) method as depicted in Fig. 3. Among 98 sequences, 31 sequences were taken as representative group for the comparative analysis for cultivar and wild samples from the laboratory source. Sequences for the subspecies and hybrids were obtained from the GenBank and the sequence of the species *Ensete ventricosum* was treated as an outgroup. The phylogenetic tree (Fig. 3) consists of three main clades, viz., A, B, and C. In clade A, cultivar red banana was found to be evolutionarily related to wild species, viz., *M. balbisiana*, *M. textilis* and *M. schizocarpa.* Further the cultivar red banana was found to be closer to subspecies *M. acuminata* subsp. *truncata*. The clade B consisted of 5 cultivars, viz., *Pisang lilin*, *M. acuminata var. flava isolate,* chemmatti, grandnain and nadan and all those cultivars were found to be closely related to the banana subspecies, viz., *M. acuminata* subsp. *microcarpa* and *M. banksii*. Among the 4 cultivars belonging to clade C, 3 cultivars, viz., *Njalipoovan, Matti* and *Kunnan,* were found to be closely related to wild species of banana, viz., *M. acuminata, M. balbisiana* and *M. accuminatavar. Burmanicoides,* respectively.

**Fig. 4:**
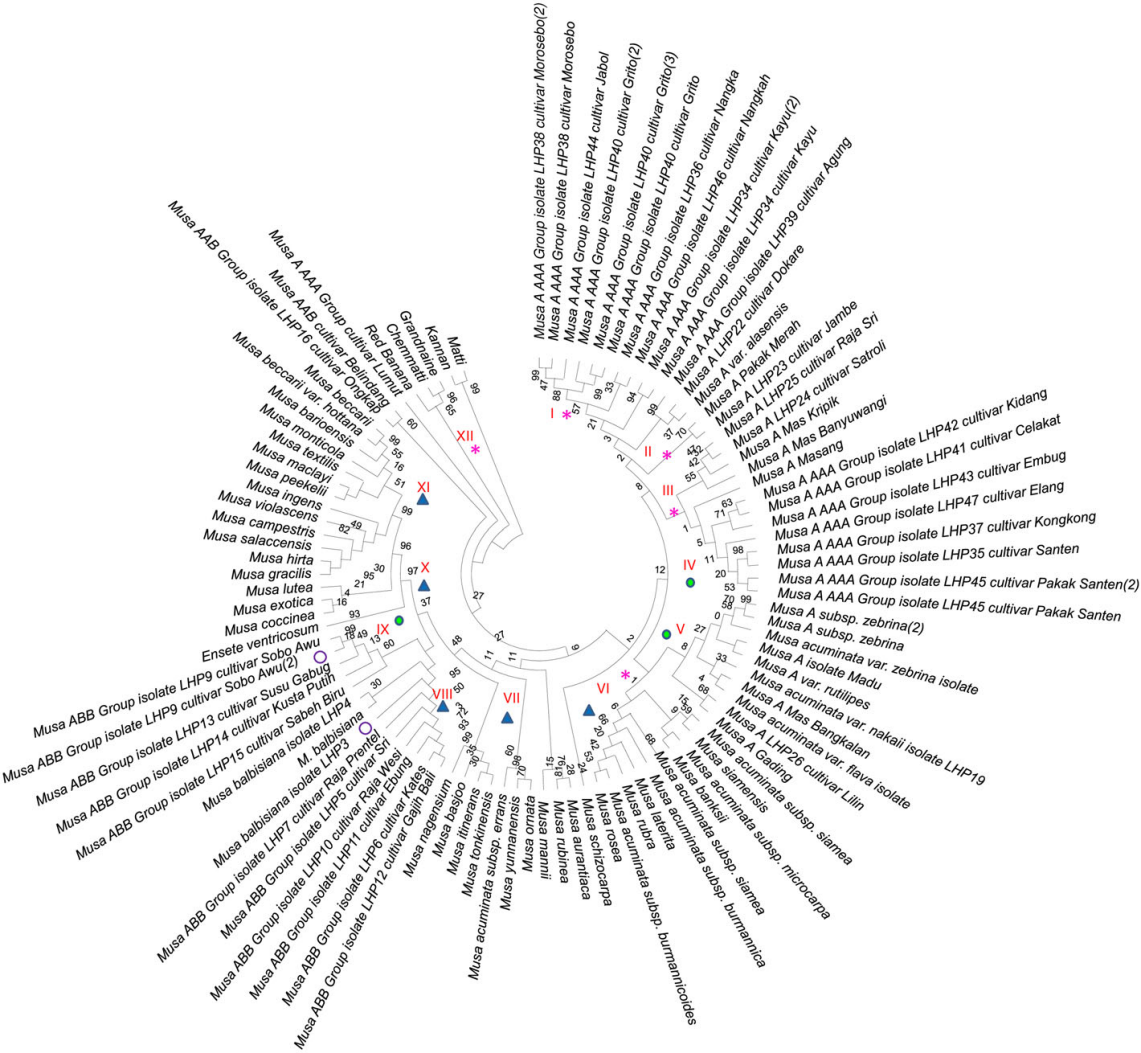
Maximum Likelihood (ML) tree based on ITS2 sequences for *Musa* species. Numbers above branches indicate bootstrap support (BS ≥ 50) values.  - Wild and subspecies of *Musa,* * - Cultivar species of *Musa,* − Varieties of *Musa*,  -Outgroup

From (Fig. 4), it could be inferred that the clade I was the most complex with 50 cultivars belonging to the wild species *Musa balbisiana*. Six banana species, viz., *M. violascens, M. splendida, M. hirta, M. campestris, M. gracilis* and *M. salaccensis*, were grouped into the same clade I. The species *M. beccarii,* and its variety were assigned to clade II. The species *M. peekelii* and *M. ingens* are distinct from the species *M. maclayi,* which exhibited high similarity index and were placed in the clade III. The species *M. monticola* and *M. barioensis* were placed in clade IV due to their closeness. Four other species, viz., *M. textilis, M. jackeyi, M. peekelii* and *M. troglodytarum* were grouped under clade V and the species *M. coccinea* and *M. lutea* were placed in the neighboring section of clade V. The following species, viz., *M. rosea, M. serpentina, M. rubra, M. laterita, M. textilis* were found to share same allelic profiles with the wild species *M. balbisiana* and *M*. *accuminata*. The following two species, viz., *M. zaifei, M. siamensis* and also the different varieties collected by us, viz., *Robusta, Red banana, cultivar Pisang lilin, Nadan, Chemmatii* and *Grandnain* shared a comparable allelic profile with the wild species - *M. accuminata*. Yet another group of seven species, viz., *M. mannii, M. ornata, M. yunnanensis, M. tonkinensis, M. itinerans, M. formosana* and *M. viridis* were grouped under the same section and exhibited closer relationship with the wild species *M. balbisana*. A single cultivar was separated from the wild species *M. balbisana* and was elevated as a species, viz., *M. nagensium.* Clade II consisted of 5 taxon which included four cultivars, viz., *Njalipoovan, Matti, Kunnan* and *wild M. accuminata var burmanicoides AA,* shared significant genetic relationship with wild species of *M. accuminata*. Clade III consisted of a single taxon - *M. basjoo,* which is found to be distinct from the wild species, viz., *M. accuminata* and *M. balbisiana* as shown in Fig. 4.

### Data analysis using restriction enzymes

Results of the present study showed that the restriction enzymes - *MseI* and *Ava*II*,* provided the best discriminatory power to differentiate the haploids of *Musa* species using ITS2 sequences. *MseI* showed single restriction site for 11 genomes of wild and cultivars of banana. A second group of 3 genomes showed two restriction sites at different locations on the genome. *Ava*II showed one, two and three restriction sites in the II group of 3 genomes, respectively.

## Discussion

A common problem for banana researchers and horticulturists in Southeast Asia is the presence of numerous cultivar names and synonyms in different languages in the region. Having a better knowledge of synonyms may promote banana trade and commerce. A rapid and reliable method for species and cultivar recognition is vital to certify the fruits and plantlets of *Musa* sp. and to preserve banana germplasm resources [9]. To our knowledge, this is the first report wherein DNA barcoding has been employed in the identification of different species and cultivars of *Musa* using a large sample size. An ideal DNA barcode should include higher inter-specific but low intra-specific divergence to discriminate different species [24] which has been shown in many earlier studies for various species and cultivars of *Colletotrichum* isolates [41], fig [42], grapevine [43], and pineapple [44]. In the present study, ITS2 was found to possess a sufficient variable region between the different species and cultivars for the determination of genetic divergence with high discriminatory ability. Morphological characters were found to resolve *M. acuminata* populations that were cultivated only at high land regions. In contrast, low land banana populations are known to show overlapping and no distinguishing pattern of phenotypic traits were observed [45]. PCR-RFLP of the ITS region using *Rsa*I restriction endonuclease was used on 68 banana accessions, which showed consistent and distinguishing polymorphic banding of DNA patterns between the wild species and cultivars of *M. acuminata* [40, 46]. Similarly, acceptable structural diversity and molecular phylogeny were observed when an ITS1–5.8S-ITS2 region was used for the species of Musaceae [47]*.* Based on the results of the present study, we propose that ITS2 can be an ideal DNA barcode candidate for *Musa* sp. However, it is pertinent to mention that the phylogeny of the members of the Musaceae remains still controversial. To cite a few cases, it is known that the taxonomic position of the species *M. beccarii* remains uncertain and the species *M. ingens* is still not documented unambiguously in taxonomic attributes [48]. Hence, the taxonomic assignment of cultivars of *Musa* based on the ITS2 and its discriminating power in firmly assigning the identification and nomenclature of the members of the Musaceae might prove to be conclusive.

The phylogenetic tree shows three clades, viz., A, B and C. Clade A is found to contain two clusters. The B genome of *M. balbsiana* seems to be closer to *M. acuminata* subsp. *Siamea*, whereas subsp. *burmannica* and *burmannicoides* form distinct and separate groups (Fig. 3). Based on BLAST1 and distance-based identification methods the cultivars - red banana and robusta of AAA genome was found to be closer to subspecies *M. acuminata* subsp. *truncata*. The cultivar red banana showed 99 and 99.1% as *Musa acuminata* subsp. *malaccensis*, on the basis of the above-mentioned two methodologies, respectively.

In the clade B, the species *M. textilis, M. banksii x Musa schizocarpa* and *M. schizocarpa* are grouped under the same cluster. The hybrid *M. banksii x Musa schizocarpa* was closely related to the wild species *M. schizocarpa.* The cultivar cluster - *pisang lilin* is inferred to be closely related to *M. acuminata* subsp. *microcarpa* and the subspecies *M. banksii.* Based on the BLAST1 and distance based identification methods, the A genome of cultivar *Pisang lilin* was found to show 99 and 98.9% similarity with *Musa acuminata var. zebrina,* respectively. The cultivar *chemmatti AA* and Grandnain were found to be closely related and identified as *Musa campestris* and *Musa acuminata* with a similarity of 97 and 94%, respectively. The cultivar nadan showed 96% similarity with subspecies *M. banksii*. In the clade C, the wild species of banana *M. balbisiana and M. acuminata* and the subspecies *M. accuminata var. burmanicoides AA* and the cultivar *Njalipoovan* and *Matti* of A genome were grouped under the same cluster. The cultivar *Njalipoovan* and *Matti* were identified as *Musa acuminata* subsp. *malaccensis* and *M. acuminata var. Zebrine,* respectively with high degree of certainity with the employment of barcoding methodologies. The species *M balbisiana* isolate LHP3 was grouped as *Musa ABB*. Thus, the results and inferences of the present study pinpoint that cultivars used in the present study might have originated from the wild species *M. acuminata* and its subspecies, respectively and *Ensete ventricosum* as an outgroup.

## Conclusion

In summary, our study demonstrated that ITS2 is an ideal DNA barcode (a) to identify *Musa* subspecies or cultivars and (b) for the reconstruction of the phylogeny of the genus *Musa*. However, more *Musa* species need to be included in the future to verify whether these findings hold good even if closely related taxa are newly included. In conclusion, DNA barcoding offered highly useful genetic information about very complex *Musa* species, which will be very useful for germplasm management and in resource protection.

## Methods

### Plant materials

From GenBank, 321 sequences were obtained, out of which 256 sequences belong to 46 species/subspecies and 65 sequences to wild isolates. We sequenced 28 *Musa* samples out of which 12 species were from the Western Ghats of India. Those sequences were submitted to GenBank and their accession numbers are given in Table 5 and their images are shown in supplementary Fig. 1. Nearly 46 annotated species and subspecies of GenBank sequences were employed in the present study and are shown in supplementary Table 1.

**Table 5:** Number of chromosomes and ploidy levels of *Musa* species */*Cultivar names and its GenBank Accessions

S.NO	Accession Number	Species/ Cultivar Names	Number of Chromosomes	Ploidy	Cultivated Clones/ Subspecies
1	KY710751	*cultivar Nadan*	33	3n = 3x	Cultivated clones
2	KY710752	*Musa acuminata* subsp. *burmannicoides*	22	2n = 2x	Subspecies
3	KY710753	*Musa acuminata*	22	2n = 2x	Wild Species
4	KY710754	*cultivar Njalipoovan*	33	3n = 3x	Cultivated Clones
5	KY710755	*Musa balbisiana*	22	2n = 2x	Wild Species
6	KY710756	*Musa acuminata*	22	2n = 2x	Wild Species
7	KY710757	*cultivar Pisang lilin*	33	3n = 3x	Cultivated Clones
8	KY710758	*cultivar Chemmatti*	33	3n = 3x	Cultivated Clones
9	KY710759	*Musa acuminata*	33	3n = 3x	Wild Species
10	KY710760	*cultivar Kunnan*	33	3n = 3x	Cultivated Clones
11	KY710761	*cultivar Njalipoovan*	33	3n = 3x	Cultivated Clones
12	KY710762	*cultivar Grandnain*	33	3n = 3x	Cultivated Clones
13	KY710763	*cultivar Matti*	33	3n = 3x	Cultivated Clones
14	KY710764	*Red Dacca (Red Banana)*	33	3n = 3x	Cultivated Clones
15	KY710765	*Robusta*	33	3n = 3x	Cultivated Clones

### DNA extraction, amplification and sequencing

Fresh, young leaves of sampled specimens were collected and genomic DNA was isolated by following [49]. The ITS2 region was amplified using the following pair of F-5′ ATGCGATAC TTGGTGTGAAT 3′ and R-5′ TCCTCCGCTTATTGATATGC 3’ of universal primers [50, 51]. Primers were synthesized by Integrated DNA Technologies, USA. PCR was carried out in 25 μL volume containing 10X PCR Buffer, 2.5 mM Mg^2+^, 0.4 mM dNTPs, 0.5 μM of each primer, 1 U Taq DNA polymerase (GeneI, India), and 30 ng genomic DNA template. The amplification was performed in a Gradient Master Cycler (Eppendorf, Germany) with a PCR program: 94 °C for 4 min, followed by 35 cycles of 94 °C for 45 s, 56 °C for 45 s, 72 °C for 1.5 min, and a final extension at 72 °C for 10 min. The PCR products were sequenced by the ABI-3130 Genetic Analyzer (Bioserve, India).

### Sequence and genetic relationship analysis

The original sequences were analyzed using MEGA [51], the ITS2 sequences were subjected to Hidden Markov Model analysis to remove the conserved 5.8S and 28S DNA sequences [24]. The ITS2 sequence was aligned using Clustal W [52] and the genetic distance computed using MEGA X 5.1 according to the Kimura 2- parameter (K2P) model [51]. The average intra-specific distance, the minimum intra-specific distance and theta prime were used to represent inter-specific divergences using the K2P model [23, 33]. The average intra-specific distance, coalescent depth and theta were calculated to evaluate the intraspecific variation [24]. The distributions of inter- versus intra-specific variability were compared using the DNA barcoding gaps [52]. Wilcoxon two-sample tests were performed as indicated previously [53]. Two methods for species identification including BLAST1 and the nearest distance method were used to evaluate the species authentication efficacy [54–56]. ITS2 sequences of *Musa* species in this study were used as query sequences. BLAST program (http://blast.ncbi.nlm.nih.gov/Blast.cgi) was used to search for the reference database for each query sequence. In the nearest distance method, correct identification means that the hit in our database based on the smallest genetic distance is from the same species as that of the query. Ambiguous identification means that several hits from our database were found to have the same smallest genetic distance to the query sequence. Incorrect identification means that the hit based on the smallest genetic is not from the expected species [33]. The discriminatory power of ITS2 sequences was calculated using MEGA X.

To understand the wild parents of cultivated bananas, the Maximum likelihood (ML) method for phylogenetic inference was carried out in MEGA version 5.1 [52], using Kimura’s 2- parameter distances [57]. Gaps were treated as missing data and bootstrap values for the ML trees were obtained from 1000 replicates. We evaluated overall nucleotide diversity and also for AAA, AAB, AA, Wild species, subspecies, and cultivar respectively. Genetic analysis of sequence polymorphism was performed using MEGA X. The number of segregating sites (S), the number of haplotypes (H), Tajima’s D was determined [58]*.* In addition, we surveyed nucleotide diversity (ℼ) [59] and theta (θ) [60] for total, silent and nonsynonymous sites independently, whereas insertion/deletions (indels) were not included in this analysis.

### Data analysis using restriction enzymes

ITS2 sequence data of 15 specimens were aligned and restriction patterns were predicted as shown in Table 3 using NEB cutter. Restriction fragments were predicted and compared for choosing the best discriminatory enzymes for haplotypes discrimination.

## Supplementary information

**Additional file 1:** **Supplementary Table 1.** Number of taxa used in this study.

**Additional file 2:** **Figure S1.** Different varieties of banana used in this study.

## Data Availability

All the data analyzed in this study are included in this article and its supplementary information files with accession numbers at the NCBI database (https://www.ncbi.nlm.nih.gov/).

## Abbreviations

ITS: Internal transcribed spacer
matK: maturase K
MEGA: Molecular Evolutionary Genetics Analysis
ML: Maximum Likelihood
K2P: Kimura 2- parameter
