# Supplementary material for: Molecular identification and evolutionary relationships between the subspecies of Musa by DNA barcodes
Source: BMC Genomics. 2020 Sep 24;21:659. doi: 10.1186/s12864-020-07036-5 (PMC7513480; doi:10.1186/s12864-020-07036-5)
Supplement: Supplementary file 1 — Additional file 1: Supplementary Table 1. Number of taxa used in this study. [file 12864_2020_7036_MOESM1_ESM.docx]

**Table S1** Number of taxa used in this study

| S. No | Species/Sub species  Name | Number of  Isolates | Voucher  Number | Collection Country | GenBank Accession  Number |
| --- | --- | --- | --- | --- | --- |
| 1 | *M. acuminata* | 51 | LHP33 | Indonesia | KT696474 |
| 2 | *M. balbisiana* | 23 | LHP4 | China | FJ428102 |
| 3 | *M. nagensium* | 3 | GXJ562 | China | JF977090 |
| 4 | *M. basjoo* | 2 | Nil | China | FJ428100 |
| 5 | *M. tonkinensis* | 1 | Nil | China | FJ428099 |
| 6 | *M. itinerans* | 7 | GXJ533 | China | JF977084 |
| 7 | *M. ornate* | 1 | Nil | China | FJ428096 |
| 8 | *M. yunnanensis* | 9 | GXJ558 | China | FJ428095 |
| 9 | *M. rubinea* | 3 | GXJ549 | China | FJ428093 |
| 10 | *M. velutina* | 6 | GXJ557 | China | FJ428092 |
| 11 | *M. mannii* | 7 | GXJ540 | China | KJ847172 |
| 12 | *M. aurantiaca* | 2 | GXJ564 | China | FJ428090 |
| 13 | *M. schizocarpa* | 5 | 0846 | China | FJ428088 |
| 14 | *M. siamensis* | 1 | GXJ501 | China | KJ847169 |
| 15 | *M. laterita* | 4 | GXJ539 | China | FJ428082 |
| 16 | *M. rubra* | 1 | GXJ543 | China | KJ847175 |
| 17 | *M. rosea* | 1 | GXJ542 | China | KJ847174 |
| 18 | *M. ingens* | 1 | GXJ576 | China | KJ428076 |
| 19 | *M. campestris* | 2 | GXJ525 | China | FJ626367 |
| 20 | *M. gracilis* | 1 | SS&JS46 | Thailand | KT257616 |
| 21 | *M. hirta* | 1 | GXJ531 | China | KJ847171 |
| 22 | *M. monticola* | 1 | GXJ541 | China | KJ847173 |
| 23 | *M. salaccensis* | 1 | GXJ552 | China | KJ847179 |
| 24 | *M. violascens* | 1 | GXJ546 | China | KJ847178 |
| 25 | *M. peekelii* | 1 | 0917CON2 | NA | HQ331366 |
| 26 | *M. textilis* | 4 | GXJ569 | China | JF977096 |
| 27 | *M. maclayi* | 5 | GXJ573 | USA | JF626369 |
| 28 | *M. barioensis* | 0 | NA | China | FJ428067 |
| 29 | *M. beccarii* | 4 | GXJ523 | NA | JF428064 |
| 30 | *M. lutea* | 0 | NA | NA | NA |
| 31 | *M. coccinea* | 4 | GXJ529 | China | JF977078 |
| 32 | *M. acuminata x M. schizocarpa* | 6 | 0954CON2 | NA | HQ331426 |
| 33 | *M. banksii x M. schizocarpa* | 1 | 1014con1 | NA | HQ331425 |
| 34 | *M. acuminata x M. textilis* | 4 | 1213CON4 | NA | HQ331419 |
| 35 | *M. zaifui* | 1 | GXJ578 | China | KJ847185 |
| 36 | *M. chunii* | 1 | GXJ577 | China | KJ847184 |
| 37 | *M. troglodytarum* | 1 | GXJ57 | China | KJ847182 |
| 38 | *M. ornata* | 6 | GXJ565 | China | FJ626382 |
| 39 | *M. tonkinensis* | 1 | GXJ544 | China | KJ847176 |
| 40 | *M. viridis* | 1 | GXJ54 | China | KJ847167 |
| 41 | *M. jackeyi* | 1 | 0588con1 | NA | HQ331362 |
| 42 | *M. splendida* | 1 | NA | China | FJ626386 |
| 43 | *M. formosana* | 1 | NA | China | FJ626379 |
| 44 | *M. sanguinea* | 1 | NA | China | FJ626378 |
| 45 | *M. paracoccinea* | 2 | GXJ566 | China | FJ626375 |
| 46 | *M. serpentina* | 1 | SS&JS 246 | Thailand | KT257620 |
