# Supplementary figures and images for: Molecular identification and evolutionary relationships between the subspecies of Musa by DNA barcodes
Source: BMC Genomics. 2020 Sep 24;21:659. doi: 10.1186/s12864-020-07036-5 (PMC7513480; doi:10.1186/s12864-020-07036-5)

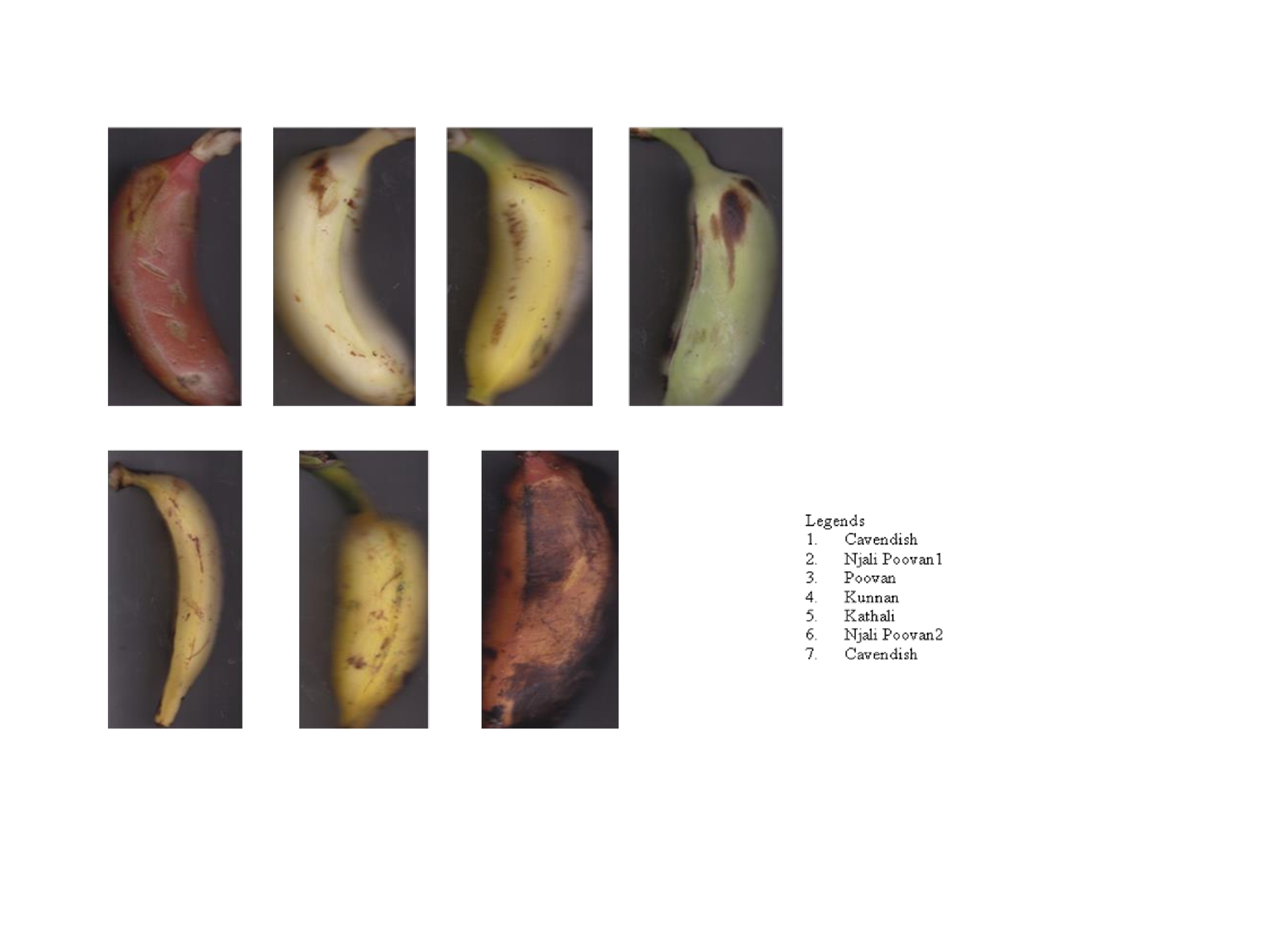

Supplement: Supplementary file 2 — Additional file 2: Figure S1. Different varieties of banana used in this study. [file 12864_2020_7036_MOESM2_ESM.tiff]
